# Detection of Retinal Nerve Fiber Layer Defects in Alzheimer’s Disease Using SD-OCT

**DOI:** 10.3389/fpsyt.2014.00022

**Published:** 2014-02-25

**Authors:** Robert Kromer, Nermin Serbecic, Lucrezia Hausner, Lutz Froelich, Fahmy Aboul-Enein, Sven C. Beutelspacher

**Affiliations:** ^1^Department of Ophthalmology, Medical Faculty Mannheim, Ruprecht-Karls-University Heidelberg, Mannheim, Germany; ^2^Division of Geriatric Psychiatry, Central Institute of Mental Health, Medical Faculty Mannheim, Ruprecht-Karls-University Heidelberg, Mannheim, Germany; ^3^Department of Neurology, SMZ-Ost Donauspital, Vienna, Austria

**Keywords:** Alzheimer’s disease, optical coherence tomography, retinal nerve fiber layer, retinal nerve fiber layer thickness, dementia

## Abstract

**Introduction:** Our aim is to examine the clinical value of spectral-domain optical coherence tomography (Spectralis OCT) to detect retinal nerve fibre layer defects in patients with clinically defined Alzheimer‘s disease (AD).

**Material and Methods:** This cross-sectional study included 22 patients with AD (mean age: 75.9 ± 6.1 years) and 22 healthy age- and sex-matched controls. Neuro-ophthalmologic examinations and a series of high-resolution OCT examinations of the peripapillary retinal nerve fiber layer (RNFL) thickness using the Spectralis 3.5-mm circle scan protocol with ART-Modus and eye tracking were obtained, and compared to age- and sex-matched healthy control subjects.

**Results:** Patients with AD showed a significant decrease in RNFL thickness in the nasal superior sector compared to the control group (101.0 ± 18.18 μm versus 122.8 ± 28.08 μm; *P* < 0.0001). In all other sectors, independently of disease duration, no significant difference in RNFL thickness compared to controls was detected. Using the advanced age- and gender-matched measurement model, 32 out of 42 eyes (76.19%) as pathologic with 67 abnormal sectors were detected.

**Discussion:** As examined by spectral-domain OCT, patients with mild to moderate stages of AD showed a significant reduction of RNFL thickness in the nasal superior sector. Nevertheless, successive studies are needed.

## Introduction

Alzheimer’s disease (AD) is a long term progressive neurodegenerative disease with a large variability between subjects (1). The pathology of AD may evolve in the brain years before clinical dementia, i.e., progressive loss of memory and other cognitive functions, associated with functional decline and behavioral disturbances, is recognized. Thus, a major research focus is on diagnosing AD in the stage of mild cognitive impairment (MCI), i.e., when gradual impairment of memory and other cognitive function develops that precedes the point at which significant interference in daily activities occurs. To support the clinical diagnosis at this heterogeneous clinical stage, biomarkers have been validated and included into the clinical diagnostic criteria (2, 3), which consist of magnetic resonance imaging (MRI) to show the atrophy of the medial temporal lobe (4, 5), positron emission tomography (PET) to detect changes in the metabolism of glucose (5, 6) and Aβ deposition in the brain (7, 8) as well as the cerebrospinal fluid analysis (CSA) to measure tau protein and Aβ peptide levels (9, 10).

A novel ophthalmic imaging method, optical coherence tomography (OCT), has been discussed to be useful for the detection of early stages of AD (11–13). OCT allows for a non-invasive visualization of axons of the central nervous system (CNS) providing cross-section imaging of the retina and is able to measure the retinal nerve fiber layer thickness (RNFLT) (14, 15). It has been used for the diagnosis of many retinal and optic nerve diseases including the glaucomas (16–23). The human eye is an embryological protrusion of the brain, and the nerves and axons of the retinal nerve fiber layer (RNFL) are similar to those in the brain. An intriguing hypothesis has been proposed that the neurodegenerative process in AD may also affect the RNFL of the eye as well. Years ago, histopathological studies suggested that the RNFL was not affected by AD (24, 25). In contrast, previously published OCT studies carried out by traditional time-domain OCT (TD-OCT) devices claimed to be able to detect a loss of the RNFL in patients with AD (12, 13, 26–28). The observed reduction in RNFLT was evident in MCI and could therefore be used to discriminate between different stages of AD (11–13). It was shown that RNFLT was reduced in particular in the superior quadrant of the peripapillary retina (26), in addition to changes in macula thickness and volume (12).

However, conventional TD-OCT technology suffers from technical inaccuracy due to a lack of exact scan centering around the optic disk as well as relatively slow scanning speed during image acquisition (20, 22). In contrast, latest advances in high-resolution spectral-domain OCT (SD-OCT) technology providing dramatically increased scanning speed and image resolution combined with eye-tracking capability already proved to be very helpful in understanding the observed changes in RNFLT more accurately thereby avoiding data misinterpretation in the context of complex neurodegenerative disorders (20, 23, 27).

In order to evaluate the feasibility und usefulness of latest SD-OCT technology in the diagnostics of AD, we carried out a preliminary study: (i) to identify a potential RNFLT reduction in AD patients, (ii) to detect whether this potential RNFLT change is global or sectoral in nature, and (iii) to compare the effect of the RNFLT change to SD-OCT standard values provided by the manufacturer and to age- and sex-matched controls.

## Materials and Methods

For this study, patients with mild to moderate AD and a cognitively healthy age-matched control subjects were recruited from the Memory Clinic of the Department of Geriatric Psychiatry of the Central Institute of Mental Health, Mannheim, Germany. The research protocol was approved by the Ethics committee II of the Medical Faculty Mannheim, Ruprecht-Karls-University Heidelberg, Mannheim, Germany and followed the recommendations of the Declaration of Helsinki. Written informed consent was obtained from each patient, or his/her caregiver (spouse or adult child), if the patient was incompetent of giving informed consent and from each comparison subject before any examination procedures were performed.

A physician cooperating with a multi-professional team (neurologists, psychiatrists, psychologists) performed and utilized diagnostic assessments at the Memory Clinic. The diagnosis of mild to moderate AD was given to all patients after a detailed medical history from patient and caregiver; general physical, neurological, and psychiatric examinations demonstrating progressive cognitive decline which interfered with everyday functioning and the absence of any neurological or psychiatric disorder possibly causing dementia (except for AD). Laboratory testing comprising a CBC with differential counts, syphilis screening, and measures of serum electrolytes, liver and renal function, cholesterol status, thyroid function, and serum vitamin B12 and folate levels were done to exclude secondary causes of dementia. Detailed neuropsychological tests (CERAD test battery WMS-LM, TMT-A, and -B) provided evidence of cognitive impairment in two or more clinical domains severe enough to cause impairment in activities of daily living. A structural MRI scan of the brain was performed in all subjects and, by visual rating, showed evidence of medial temporal lobe atrophy in the absence of major white matter abnormalities and/or other cerebrovascular disorder. In the majority of patients, CSF analysis revealed a reduction of a-beta42 peptide and increase of tau and/or phospho-tau protein levels typical for AD. The study investigated 42 eyes of 22 patients with mild to moderate AD (mean age: 75.9 ± 6.1 years; 14 women) and 42 eyes of 22 age- and sex-matched healthy controls. Two subjects in either group had only one eye. The ophthalmologic inclusion criteria were: (i) best-corrected visual acuity of 0.3 LogMAR or better, (ii) spherical refraction within ±5.0 diopters (D), (iii) cylindrical correction within ±2.0 D, and (iv) normal results for visual field testing (Swedish Interactive Thresholding Algorithm SITA; Octopus 101 Perimeter; Haag-Streit Deutschland GmbH, Wedel, Germany). The patients’ history and available medical records were reviewed for diseases possibly reducing the RNFLT. Patients with intake history of alcohol abuse, a body mass index >30, and diseases that could reduce RNFL thickness were excluded from the study. We also excluded patients with an intraocular pressure ≥21 mmHg, history of glaucoma, anterior ischemic optic neuropathy, high myopia, prior ocular surgery, and congenital abnormalities of the optic nerve.

Patients and control subjects underwent various ophthalmic examinations: (i) assessment of best-corrected visual acuity by auto-refractometry (auto-refractometer (OCULUS/NIDEK auto-refractometer, OCULUS Optikgeräte GmbH, Wetzlar, Germany) followed by subjective refractometry using the ETDRS (Early Treatment of Diabetic Retinopathy Study) 2000 chart for high-contrast visual acuity, (ii) slit lamp assisted biomicroscopy of the anterior segment, (iii) ophthalmoscopy after medical dilation of the pupil, (iv) visual field testing (Swedish Interactive Thresholding Algorithm SITA; Octopus 101 Perimeter; Haag-Streit Deutschland GmbH, Wedel, Germany), (v) Goldmann applanation tonometry, (vi) SD-OCT for RNFL thickness measurement.

The thickness of the RNFL was measured by SD-OCT (Heidelberg Spectralis OCT, SPECTRALIS software version 5.3.3.0, EYE EXPLORER Software 1.6.4.0; Heidelberg Engineering, Heidelberg, Germany). It obtains non-contact frames in high resolution of the RNFL. The device is a combination of conventional OCT technology and confocal scanning laser ophthalmoscopy. A super luminescence diode is used to emit a laser scan beam with the wavelength of 870 nm. The SD-OCT can receive up to 40,000 A-scans per second with a depth resolution of 7 μm and a transversal resolution of 14 μm. The cSLO uses a laser in order to illuminate the retina and to scan it point by point to deliver a real-time capture of the retina. This reference image is linked and saved to the SD-OCT scan with an eye-tracking system (TrueTrack™, Heidelberg Engineering, Heidelberg, Germany). Based on eye, an additional feature – the automatic real-time averaging mode (ART) – results in even higher quality. First the area of interest is found with cSLO and then locked. Every time the eye is tracked in the same direction B scans are taken. Measured data are automatically averaged and artifacts are minimized. In this study, only high quality data with at least a total of 18 frames were used to provide the most accurately result as possible. Due to high-resolution scans, the individual layers of the RNFL were discriminable – even without pupil dilatation. We first positioned the optic disk perfectly centered and enabled the ART mode. For each patient, three high resolution and three high-speed scans were acquired by one examiner to minimize the variability. All images not reaching our criteria of quality were dismissed: (i) the fundus had to clear before and during image acquisition, (ii) absence of scan and algorithm failures, (iii) the gray scale saturation of each RNFL needed to be consistent with a maximum shading of the retinal pigment epithelium, and (iv) no discontinuation of the scanned layer.

Statistical analysis was carried out using a commercially available software package (Prism 5 for Mac OSX; GraphPad Software, Version 5.0c). Mean and standard deviations were presented. Study and control group were compared with each using one-way ANOVA with Bonferroni’s Multiple Comparison Post Test. All *P*-values were two-tailed and a P-value < 0.05 was considered to indicate statistical significance.

## Results

The study included 22 patients with AD (mean age: 75.9 ± 6.1 years; age range: 66–88 years; 14 women) and 22 healthy controls (mean age: 64.0 ± 8.2; age range: 53–85 years; 15 women) matched for age and gender with the patients of the study population.

**Figure 1:**
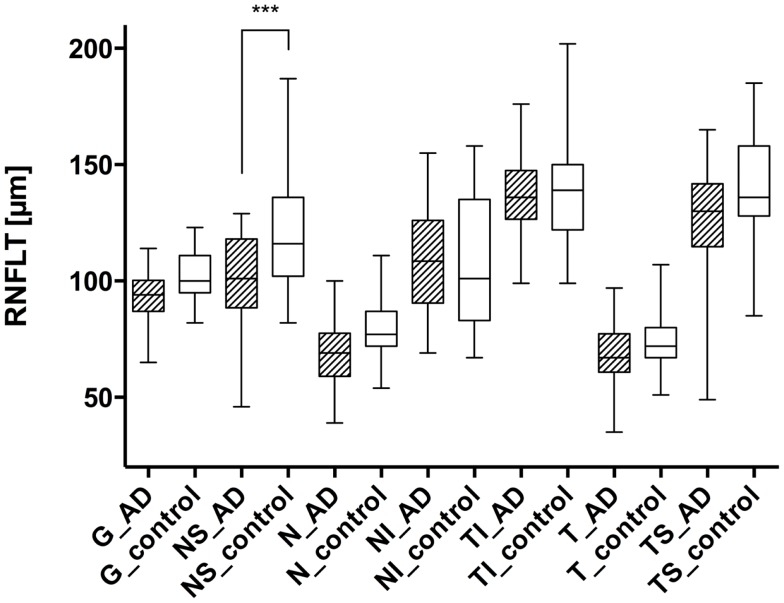
**Comparison of the retinal nerve fiber layer thickness in different peripapillary sectors: each left column represents the patient with AD (AD), while the right columns show the values of the control group (control)**. The different sectors are labeled as follows: global (G), nasal superior (NS), nasal (N), nasal inferior (NI), temporal inferior (TI), temporal (T), and temporal superior (TS). Significant differences (with Bonferroni correction) were only registered in the nasal superior sector (*P* < 0.0001), indicated as ***.

**Figure 2:**
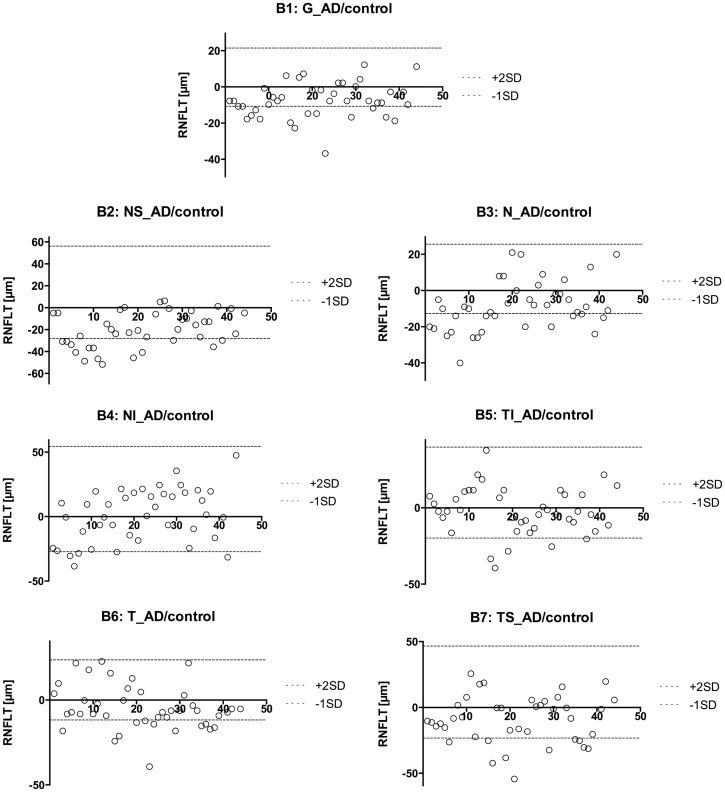
**Presentation of the retinal nerve fiber layer thickness values of the AD eyes compared to the mean of the control eyes**. The different sectors are labeled as follows: global (G, B1), nasal superior (NS, B2), nasal (N, B3), nasal inferior (NI, B4), temporal inferior (TI, B5), temporal (T, B6), and temporal superior (TS, B7). A circle represents the AD eyes. This way, we were able to identify patients with an RNFL increase or atrophy. We found 67 abnormal sectors and 32 eyes with pathologic RNFLT (out of 42 eyes).

**Figure 3:**
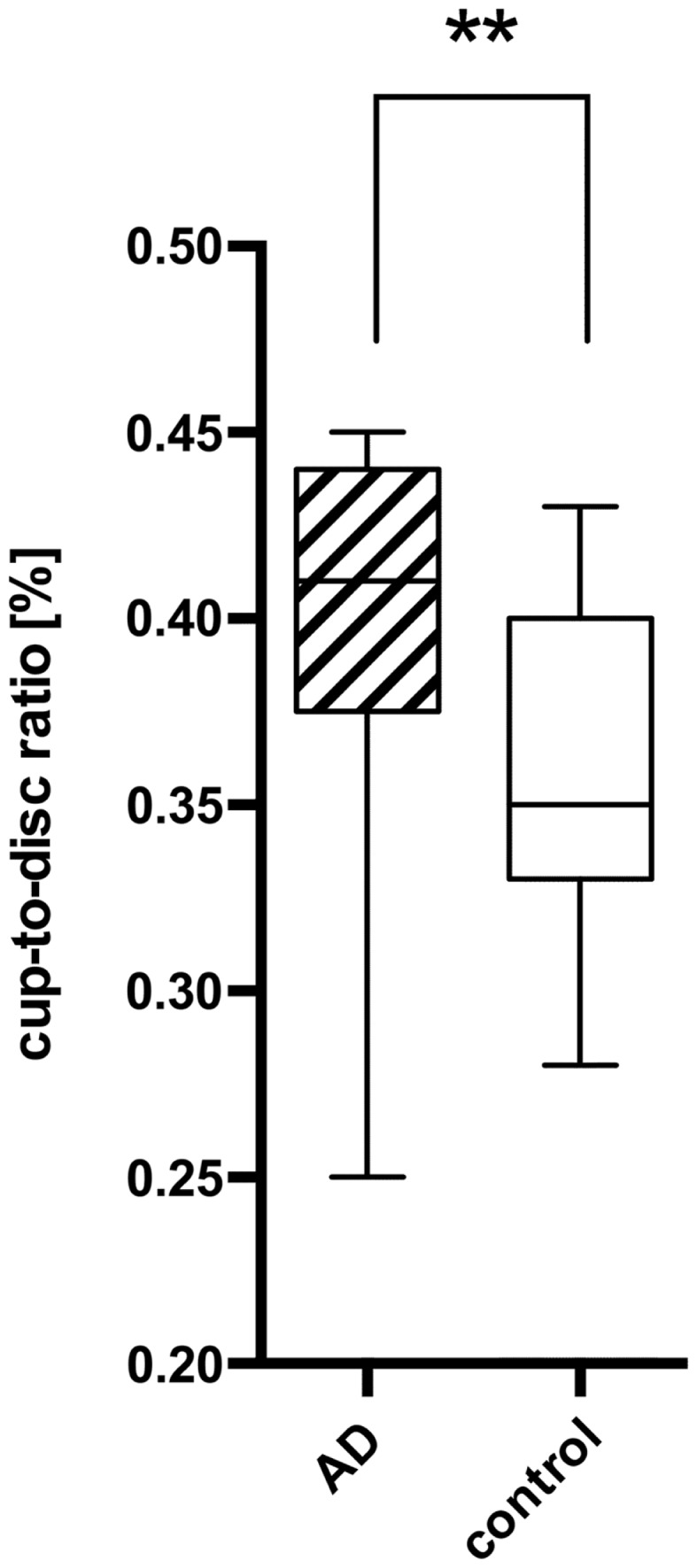
Comparison of the cup-to-disk ratio (CD-ratio) between AD patients (AD) and controls (control) with a significant difference (*P* = 0.001) indicated as **. The mean CD-ratio of AD patients was 0.396, the one of the control patients 0.359.

Using the standard normative database of the SD-OCT device, the RNFLT measurements compared for each patient of the study group showed no RNFLT reduction neither globally nor in any of the peripapillary sectors (P > 0.05) except for one peripapillary sector of one eye where the SD-OCT software identified a significant reduction in RNFLT (Table 2, Figures 1 and 2). The mean MMSE score was 22.59 ± 5.47 in the AD group. The RNFL thickness measurements compared for each patient (Table 1) showed a RNFLT reduction of the nasal superior sector in the AD group (P < 0.0001 by ANOVA using Bonferroni’s correction for multiple comparisons). Every other sector and global RNFLT was not significant different. The P-value for the temporal superior sector showed a tendency of significance (P = 0.0349) after Bonferroni correction for multiple comparisons. In all other cases, the P-value was clearly above 0.05. Neither age or gender nor known disease duration or AD stage was significantly correlated with RNFLT. Alzheimer patients had a significantly increased cup-to-disk ratio (CD-ratio) compared with controls (*P* = 0.001, Figure 3). However the CD-ratio did not correlate with the RNFLT.

**Table 1:** **Comparison of the different sectors around the optic disk regarding the retinal nerve fiber layer thickness**.

Multiple comparison post test	Mean diff.	*t*-Ratio	P-value	95% CI of difference
G_AD versus G_control	−7.553	1.874	>0.05	−18.43 to 3.328
NS_AD versus NS_control*	−21.79	5.407	<0.0001	−32.67 to −10.91
N_AD versus N_control	−8.594	2.132	>0.05	−19.47 to 2.287
NI_AD versus NI_control	0.8693	0.2157	>0.05	−10.01 to 11.75
TI_AD versus TI_control	−2.276	0.5646	>0.05	−13.16 to 8.605
T_AD versus T_control	−4.875	1.210	>0.05	−15.76 to 6.006
TS_AD versus TS_control	−11.28	2.798	0.0349	−22.16 to −0.3972

The table shows the mean difference (Mean diff.), the *t*-ratio, the P-value, and the 95% confidence interval (95% CI). The different sectors are labeled as follows: global (G), nasal superior (NS), nasal (N), nasal inferior (NI), temporal inferior (TI), temporal (T), and temporal superior (TS). The one-way ANOVA with Bonferroni’s Multiple Comparison Post Test showed significant lowering (marked with *) in the nasal superior sector and the temporal superior sector for AD patients. Due to Bonferroni correction for multiple comparisons, only the nasal superior sector is different. All other sectors were not significantly different.

**Table 2:** **Comparison of the different sectors around the optic disk regarding the retinal nerve fiber layer thickness**.

Patient	Mean	NS	*N*	NI	TI	T	TS
1	90.0 ± 8.5	75.0 ± 1.4	46.0 ± 9.9	111.5 ± 21.9	143.5 ± 9.2	73.0 ± 1.4	153.0 ± 17.0
2	102.0 ± 8.5	105.5 ± 3.5	60.5 ± 6.4	112.0 ± 7.1	166.5 ± 13.4	77.5 ± 17.7	157.5 ± 0.7
3	100.0 ± 0.0	99.0 ± 4.2	99.5 ± 0.7	127.5 ± 2.1	130.5 ± 2.1	61.5 ± 0.7	122.5 ± 0.7
4	99.0	124.0	92.0	127.0	134.0	58.0	108.0
5	110.0 ± 5.7	116.5 ± 4.9	81.0 ± 5.7	129.0 ± 4.2	148.5 ± 2.1	86.5 ± 13.4	151.0 ± 5.7
6	95.5 ± 4.9	110.5 ± 16.3	66.0 ± 2.8	91.5 ± 21.9	143.5 ± 23.3	68.0 ± 1.4	148.5 ± 14.8
7	108.0 ± 1.4	111.5 ± 16.3	87.0 ± 0.0	125.5 ± 4.9	147.5 ± 3.5	77.5 ± 4.9	139.0 ± 0.0
8	85.0 ± 1.4	85.5 ± 4.9	55.0 ± 1.4	73.0 ± 5.7	129.0 ± 9.9	81.5 ± 20.5	118.5 ± 7.8
9	90.5 ± 2.1	91.5 ± 7.8	67.0 ± 2.8	80.5 ± 2.1	147.0 ± 4.2	66.0 ± 0.0	139.0 ± 11.3
10	93.0 ± 0.0	110.0 ± 0.0	66.5 ± 0.7	124.0 ± 5.7	141.5 ± 7.8	59.5 ± 0.7	114.5 ± 0.7
11	91.5 ± 9.2	115.5 ± 17.7	65.0 ± 8.5	119.0 ± 5.7	119.0 ± 8.5	60.0 ± 5.7	126.0 ± 26.9
12	113.0	118.0	99.0	155.0	153.0	69.0	145.0
13	94.0 ± 0.0	118.0 ± 0.0	58.5 ± 0.7	82.0 ± 1.4	143.5 ± 3.5	81.0 ± 4.2	128.5 ± 0.7
14	97.5 ± 4.9	78.5 ± 10.6	61.5 ± 12	109.0 ± 11.3	154.5 ± 7.8	94.5 ± 3.5	124.5 ± 10.6
15	87.0 ± 0.0	79.5 ± 3.5	75.5 ± 4.9	91.0 ± 2.8	116.5 ± 9.2	83.0 ± 5.7	93.0 ± 11.3
16	103.0 ± 1.4	120.5 ± 12	79.5 ± 3.5	137.5 ± 7.8	134.0 ± 0.0	67.5 ± 0.7	139.0 ± 1.4
17	91.0 ± 0.0	92.0 ± 0.0	71.5 ± 3.5	112.5 ± 7.8	134.0 ± 2.8	61.0 ± 7.1	126.0 ± 1.4
18	136.5 ± 27.6	145.0 ± 53.7	117.5 ± 20.5	179.0 ± 12.0	204.0 ± 6.4	81.5 ± 20.5	165.5 ± 65.1
19	125.5 ± 1.4	136.5 ± 4.2	90.0 ± 10.6	145.5 ± 12.7	182.0 ± 3.5	93.5 ± 5.7	173.5 ± 7.1
20	137.0 ± 2.8	149.5 ± 7.8	102.0 ± 6.4	139.5 ± 10.6	194.5 ± 1.4	103.5 ± 2.1	200.5 ± 5,7
21	120.0 ± 2.1	170.5 ± 15.6	98.5 ± 1.4	130.5 ± 14.8	151.5 ± 4.2	78.0 ± 2.1	154.0 ± 12.0
22	141.0 ± 0.0	151.5 ± 17.0	108.0 ± 2.1	162.5 ± 15.6	198.0 ± 10.6	98.0 ± 5.7	204.5 ± 16.3
Controls	101.8 ± 10.7	122.8 ± 28.1	79.0 ± 12.8	107.5 ± 27.2	138.4 ± 19.9	74.3 ± 11.8	139.3 ± 23.3
Heidelberg spectralis values (P < 0.01)	94.5 ± 20.5	71.5 ± 33.5	102.0 ± 44.0	103.0 ± 52.0	135.0 ± 43.0	69.5 ± 27.5	129.0 ± 38.0

*The table shows the mean of both eyes and SD interval for every AD patient (in case the patient has only one eye SD is not presented). At the bottom of the table means of the control patients as well the normative data of the manufacture (kindly provided) are viewed. The different sectors are labeled as follows: global (G), nasal superior (NS), nasal (N), nasal inferior (NI), temporal inferior (TI), temporal (T), and temporal superior (TS)*.

However, to compare the RNFLT globally and in each sector further in more detail, we used a modified model described earlier (29). Namely, we plotted every measured value for each eye with a different plot for each sector (Figure 2). We used the calculated mean from the control group to show the difference between AD patients and our sex-, and age-matched healthy control group. As previously described (30), there is a normal inter-individual variation in RNFLT, above +2SD increase of RNFLT compared to reported RNFLT normal values, and the values below −1SD as RNFL decrease or atrophy. Those two borders (+2SD and −1SD) were also plotted to show deviations. This way, we were able to identify patients with RNFL atrophy and measure the affected sectors (Table 3). Our age- and sex-matched-based model marked 32 out of 42 eyes (76.19%) as pathologic with 67 abnormal sectors. Nonetheless, despite using our advanced age-, and gender-matched measurement model no RNFL loss was evident in four patients (18.18%) and one eye of additional three patients (7.14% of total eye number).

**Table 3:** **Comparison of the different sectors around the optic disk regarding the retinal nerve fiber layer thickness (RNFLT) in the eyes of AD patients**.

Patient	MMSE	Clinical dementia rating	Disease duration (months)	Cup-to-disk ratio (left eye first)	SD-OCT calculations		Age- and sex-matched calculations	
					Left eye	Right eye	Left eye	Right eye
1	17	2	3.2	0.40, 0.43	0	0	2 Atrophic	2 Atrophic
2	27	1	2.6	0,41, 0.38	0	0	1 Atrophic	1 Atrophic
3	20	2	6.9	0.40, 0.38	0	0	1 Atrophic	1 Atrophic
4	23	1	3	0.44, –	0	0	2 Atrophic	–
5	25	0.5	4	0.44, 0.45	0	–	0	0
6	23	1	7.4	0.43, 0.40	0	0	1 Atrophic	1 Atrophic
7	25	1	11	0.38, 0.35	0	0	0	0
8	29	0.5	6.3	0.44, 0.41	0	0	3 Atrophic	4 Atrophic
9	19	2	1.2	0.35, 0.29	0	0	2 Atrophic	1 Atrophic
10	20	2	18.3	0.33, 0.28	0	0	2 Atrophic	3 Atrophic
11	27	0.5	8.7	0.40, 0.40	0	0	0	4 Atrophic
12	30	0,5	32	–, 0.35	–	0	–	0
13	25	0.5	32.2	0.33, 0.40	0	1 Swollen	1 Atrophic	1 Atrophic
14	29	0.5	22.5	0.35, 0.35	0	0	1 Atrophic	2 Atrophic
15	28	0.5	6.5	0.33, 0.31	0	1 Atrophic	3 Atrophic	2 Atrophic
16	29	0.5	24.5	0.35, 0.35	0	0	0	0
17	21	1	8.5	0.45, 0.45	0	0	2 Atrophic	1 Atrophic
18	21	1	8.0	0.44, 0.44	4 Atrophic	0	4 Atrophic	0
19	14	2	6.6	0.37, 0.42	0	0	4 Atrophic	2 Atrophic
20	21	2	7.3	0.38, 0.33	0	0	0	3 Atrophic
21	13	2	63.3	0.35, 0.41	0	0	3 Atrophic	5 Atrophic
22	11	2	7.9	0.33, 0.40	0	0	1 Atrophic	1 Atrophic
Abnormal eyes					1	2	16	16
Abnormal sectors					4	2	33	34

*The first column shows the patient number, the second displays the reached points of the MMSE, the third column the Clinical Dementia Rating score, the fourth the months since diagnosis and the fifth the cup-to-disk ratio. The two blocks following show the data as obtained from the SD-OCT-based internal database (this is plotted for left and right eye). This was put into contrast to our calculations using age- and sex-matched controls (the two following blocks). At the bottom of this table, the count of the pathologic peripapillary sectors as well as eyes is shown. Thus the SD-OCT’s internal database was not able to detect 61 abnormal sectors and 29 eyes with pathologic RNFLT compared to our age- and sex-matched based model*.

## Discussion

Our results show a significant reduction of the RNFLT in the nasal superior sector in AD patients compared to age- and sex-matched controls. Globally and in all other sectors, there was no significant change detectable. The CD-ratio was significantly increased in AD patients.

Retinal nerve fiber layer thickness in AD patients has already been examined by different techniques: histopathological studies examining the RNFL of AD patients and controls post-mortem have yielded different results: while some of them did not report changes in the RNFLT at all (24, 25), others showed significant reductions (31–35). Blanks et al. reports a loss of 25% in the ganglion cell layer (34), although amyloid deposits and neurofibrillary tangles, the typical hallmarks of AD, were not found (31, 32). Earlier studies using OCT technology provided preliminary evidence that AD decreases the RNFLT (11–13, 26, 36–38). One meta-analysis (39) claimed to show a significant decrease in global RNFLT. However, due to the selection bias of the different studies and as the global reduction in some of the included study was mostly subtle, individual peripapillary sectors of the circumferential scans were examined as well in our study. Parisi et al. and Iseri et al. found that all sectors were decreased significantly. In contrast, other studies detect a decrease only in the superior and inferior sector (13, 38), or just in the superior sector (26). Several studies have shown that the CD-ratio is increased in AD patients (33, 38), furthermore it seems that due to studies the CD-ratio correlates with the RNFLT (40).

The different findings in histopathologic studies may be explained by different post-mortem evaluation techniques or diagnostic selection criteria. The disagreement in OCT studies may evolve from divergent stages of the disease as well as technical inaccuracy of OCT studies. Furthermore, the sample sizes of the studies cited were fairly low (mean number of AD patients: 16.9 ± 7.1). There is some evidence that the RNFLT may decrease with advancing disease (12). The patients with a decrease in every quadrant (global reduction in RNFLT) had fairly low MMSE scores, ranging from 11 to 19 (36) and from 8 to 28 (27). In comparison, the patients of the study of Berisha et al. had MMSE scores between 17 and 30 (26) and experienced a decrease only in the superior sector. Furthermore, the superior sector could be used to discriminate between MCI and severe AD (13), while there was no significant difference found between the RNFLT of MCI and mild AD patients (11).

As our study included a comparatively large number of participants and used latest high-resolution SD-OCT technology, we are able to extend the current evidence. (1) Our patients did not suffer from severe AD, thus the score of MMSE did not correlate with the RNFLT. (2) The reduction of RNFLT in an isolated peripapillary sector may be the first sign of an affection of RNFL in AD, which extends over the remaining sectors (and/or globally) in the course of AD, as described by others. (3) The heterogeneity of AD may finally contribute to completely unaffected visual systems in some patients, as we could demonstrate, too.

The limitations of this study are: (i) that only patients with a limited range of severity of AD were included and (ii) that only cross-sectional measurements were done without follow-up to investigate a potential progress of the RNFLT. On the other hand the strengths of our study are: (i) that patients and controls were age- and sex-matched, (ii) high-resolution SD-OCT measurements were obtained, (iii) and the clinical diagnosis of AD was obtained in a specialized memory clinic by using highly standardized clinical criteria including neuropsychological assessments and biomarkers (MTA, CSF).

In summary, we demonstrate that mild to moderate stages of AD may be associated with a reduction of RNFLT in the nasal superior sector. However, it remains unclear whether RNFLT analysis is an appropriate method for monitoring disease progression, if all AD patients are affected. Prerequisite for the interpretations of OCT-investigations are standardized technical requirements provided by latest SD-OCT technology.

## Conflict of Interest Statement

The authors declare that the research was conducted in the absence of any commercial or financial relationships that could be construed as a potential conflict of interest.
